# Supplementary material for: Structural insight into guanylyl cyclase receptor hijacking of the kinase–Hsp90 regulatory mechanism
Source: eLife. 2023 Aug 3;12:RP86784. doi: 10.7554/eLife.86784 (PMC10400071; doi:10.7554/eLife.86784)
Supplement: Supplementary file 1. [file elife-86784-supp1.docx]

**Structural insight into guanylyl cyclase receptor hijacking of the kinase–Hsp90 regulatory mechanism**

Nathanael A Caveney^1,^*, Naotaka Tsutsumi^1,2,3^, K Christopher Garcia^1,2,^*

^1^Departments of Molecular and Cellular Physiology and Structural Biology, Stanford University School of Medicine, Stanford, CA 94305, USA

^2^Howard Hughes Medical Institute, Stanford University School of Medicine, Stanford, CA 94305, USA

^3^Present address: Graduate School of Medicine, Dentistry and Pharmaceutical Sciences, Okayama University, Okayama, Japan.

*Correspondence: ncaveney@stanford.edu, kcgarcia@stanford.edu

Material availability: The plasmids used in this study are described below and available from KCG (kcgarcia@stanford.edu) by request.

| **Reagent type (species) or resource** | **Designation** | **Source or reference** | **Identifiers** | **Additional information** |
| --- | --- | --- | --- | --- |
| Recombinant DNA reagent | pD649-GCN4-TM-GC-C_ICD (plasmid) | This paper |  | See: Methods - Cloning and protein expression |

>pD649-GCN4-TM-GC-C_ICD

ATGAAGACTATAATCGCCCTCTCCTACATATTTTGCTTGGTGTTTGCCGACTACAAGGACGACGATGATAAAGGGTCTCTGGAAGTCCTTTTCCAGGGGCCCGGTAGAATGAAGCAACTCGAAGACAAAGTCGAGGAGCTTCTCTCTAAAAACTATCATCTCGAGAATGAAGTGGCCCGCCTGAAAAAACTCGTTGGCGAACGGAAGTTGCCAAATGATATAACCGGACGCGGACCACAAATTCTTATGATAGCTGTCTTTACACTTACAGGGGCTGTGGTGCTCCTGCTGTTGGTAGCGCTCCTCATGCTTCGAAAATACAGAAAGGATTATGAGCTCCGGCAGAAGAAATGGAGTCACATTCCTCCGGAAAACATTTTTCCTCTCGAGACAAACGAGACGAACCACGTATCCCTGAAAATCGACGACGACAAAAGACGAGATACCATCCAGCGATTGCGCCAGTGTAAATACGATAAGAAAAGAGTAATCCTCAAAGATTTGAAGCATAACGATGGCAATTTCACCGAGAAACAGAAGATTGAGCTGAATAAACTCTTGCAAATTGACTATTATAATTTGACCAAGTTTTACGGGACGGTCAAGTTGGACACGATGATATTTGGGGTGATAGAATATTGTGAACGCGGTAGTCTCAGAGAAGTCCTGAATGACACGATATCCTATCCCGATGGCACCTTTATGGACTGGGAATTCAAGATTAGCGTACTGTACGATATCGCCAAAGGAATGTCATATCTCCATAGTAGCAAAACCGAGGTTCACGGTCGCTTGAAATCAACCAATTGTGTCGTAGATAGTCGCATGGTCGTGAAGATTACTGATTTTGGATGTAATTCCATATTGCCGCCCAAAAAAGACTTGTGGACCGCTCCAGAACACCTTAGGCAGGCCAACATAAGTCAAAAGGGGGACGTTTACTCCTATGGTATTATAGCACAAGAAATTATCCTCCGAAAGGAAACATTTTATACCTTGTCTTGTCGAGACAGGAACGAAAAAATTTTTCGGGTGGAGAATTCCAATGGGATGAAACCATTTAGACCCGACCTCTTTCTGGAGACAGCTGAGGAGAAAGAGCTTGAAGTTTATTTGCTGGTGAAAAACTGTTGGGAAGAGGATCCGGAAAAACGGCCCGACTTTAAGAAGATTGAGACAACCTTGGCAAAAATATTCGGGTTGTTCCATGACCAGAAGAATGAAAGCTACATGGACACATTGATTAGGAGGTTGCAACTGTACTCACGGAACCTGGAGCACCTTGTCGAGGAGAGAACGCAGTTGTATAAGGCAGAAAGGGACCGAGCAGACAGACTGAATTTTATGCTCCTCCCCAGACTGGTTGTAAAAAGCCTGAAAGAAAAAGGCTTCGTGGAGCCTGAGCTGTATGAGGAAGTGACAATATACTTCTCCGACATAGTTGGTTTCACCACTATATGCAAATATTCTACACCCATGGAGGTCGTTGATATGCTCAACGATATATACAAGTCCTTTGACCATATCGTCGATCACCATGACGTATACAAAGTGGAGACCATCGGTGACGCATACATGGTGGCGAGCGGCCTGCCTAAAAGAAACGGTAACCGACATGCGATAGACATTGCTAAAATGGCTCTGGAGATCCTTAGTTTCATGGGCACGTTCGAACTGGAGCATCTCCCAGGCCTGCCTATCTGGATTCGAATAGGTGTGCATTCCGGGCCTTGCGCGGCCGGAGTGGTTGGTATCAAGATGCCGAGATACTGTCTCTTTGGAGACACAGTTAACACCGCCTCACGCATGGAGTCTACCGGGTTGCCGCTCCGCATTCATGTAAGTGGGTCTACGATAGCGATCTTGAAACGGACAGAGTGCCAATTTCTTTACGAAGTACGCGGAGAAACATATCTCAAGGGTCGGGGTAATGAGACCACTTATTGGCTCACGGGGATGAAGGATCAGAAGTTCAATCTGCCCACTCCCCCGACCGTTGAGAACCAGCAGAGACTGCAAGCGGAATTTTCAGATATGATCGCGAACTCTCTGCAGAAGCGGCAAGCGGCTGGGATTCGCTCCCAAAAGCCTAGACGCGTTGCGTCCTATAAGAAGGGTACCTTGGAGTACTTGCAGCTGAATACAACGGACAAAGAATCAACATATTTCGCGGCAGCGCTCGAGGTACTTTTCCAAGGCCCAGGAGCTGCTGAGGATCAGGTCGATCCTCGCCTTATCGACGGCAAACACCATCATCACCATCACCACCACtaaGGATCCGTTACCCGGTAAGCCAATCGGGTATACACGGTCGTCATACTGCAGACAGGGTTCTTCTACTTTGCAAGATAGTCTTGAGTAGTAAAATAAATAGATAGAGAAAATCAGCCTCGACTGTGCCTTCTAGTTGCCAGCCATCTGTTGTTTGCCCCTCCCCCGTGCCTTCCTTGACCCTGGAAGGTGCCACTCCCACTGTCCTTTCCTAATAAAATGAGGAAATTGCATCACAACACTCAACCCTATCTCGGTCTATTCTTTTGATTTATAAGGGATTTTGCCGATTTCGGCCTATTGGTTAAAAAATGAGCTGATTTAACAAAAATTTAACGCGAATTAATTCTGTGGAATGTGTGTCAGTTAGGGTGTGGAAAGTCCCCAGGCTCCCCAGCAGGCAGAAGTATGCAAAGCATGCATCTCAATTAGTCAGCAACCAGGTGTGGAAAGTCCCCAGGCTCCCCAGCAGGCAGAAGTATGCAAAGCATGCATCTCAATTAGTCAGCAACCATAGTCCCGCCCCTAACTCCGCCCATCCCGCCCCTAACTCCGCCCAGTTCCGCCCATTCTCCGCCCCATGGCTGACTAATTTTTTTTATTTATGCAGAGGCCGAGGCCGCCTCTGCCTCTGAGCTATTCCAGAAGTAGTGAGGAGGCTTTTTTGGAGGCCTAGGCTTTTGCAAAAAGCTCCCGGGAGCTTGTATATCCATTTTCGGATCTGATCAGCACGTGTTGACAATTAATCATCGGCATAGTATATCGGCATAGTATAATACGACAAGGTGAGGAACTAAACCATGACCGAGTACAAGCCCACGGTGCGCCTCGCCACCCGCGACGACGTCCCCAGGGCCGTACGCACCCTCGCCGCCGCGTTCGCCGACTACCCCGCCACGCGCCACACCGTCGATCCGGACCGCCACATCGAGCGGGTCACCGAGCTGCAAGAACTCTTCCTCACGCGCGTCGGGCTCGACATCGGCAAGGTGTGGGTCGCGGACGACGGCGCCGCGGTGGCGGTCTGGACCACGCCGGAGAGCGTCGAAGCGGGGGCGGTGTTCGCCGAGATCGGCCCGCGCATGGCCGAGTTGAGCGGTTCCCGGCTGGCCGCGCAGCAACAGATGGAAGGCCTCCTGGCGCCGCACCGGCCCAAGGAGCCCGCGTGGTTCCTGGCCACCGTCGGCGTCTCGCCCGACCACCAGGGCAAGGGTCTGGGCAGCGCCGTCGTGCTCCCCGGAGTGGAGGCGGCCGAGCGCGCCGGGGTGCCCGCCTTCCTGGAGACATCCGCGCCCCGCAACCTCCCCTTCTACGAGCGGCTCGGCTTCACCGTCACCGCCGACGTCGAGGTGCCCGAAGGACCGCGCACCTGGTGCATGACCCGCAAGCCCGGTGCCTGACACGTGCTACGAGATTTCGATTCCACCGCCGCCTTCTATGAAAGGTTGGGCTTCGGAATCGTTTTCCGGGACGCCGGCTGGATGATCCTCCAGCGCGGGGATCTCATGCTGGAGTTCTTCGCCCACCCCAACTTGTTTATTGCAGCTTATAATGGTTACAAATAAAGCAATAGCATCACAAATTTCACAAATAAAGCATTTTTTTCACTGCATTCTAGTTGTGGTTTGTCCAAACTCATCAATGTATCTTATCATGTCTGTATACCGTCGACCTCTAGCTAGAGCTTGGCGTAATCATGGTCATTACCAATGCTTAATCAGTGAGGCACCTATCTCAGCGATCTGTCTATTTCGTTCATCCATAGTTGCCTGACTCCCCGTCGTGTAGATAACTACGATACGGGAGGGCTTACCATCTGGCCCCAGCGCTGCGATGATACCGCGAGAACCACGCTCACCGGCTCCGGATTTATCAGCAATAAACCAGCCAGCCGGAAGGGCCGAGCGCAGAAGTGGTCCTGCAACTTTATCCGCCTCCATCCAGTCTATTAATTGTTGCCGGGAAGCTAGAGTAAGTAGTTCGCCAGTTAATAGTTTGCGCAACGTTGTTGCCATCGCTACAGGCATCGTGGTGTCACGCTCGTCGTTTGGTATGGCTTCATTCAGCTCCGGTTCCCAACGATCAAGGCGAGTTACATGATCCCCCATGTTGTGCAAAAAAGCGGTTAGCTCCTTCGGTCCTCCGATCGTTGTCAGAAGTAAGTTGGCCGCAGTGTTATCACTCATGGTTATGGCAGCACTGCATAATTCTCTTACTGTCATGCCATCCGTAAGATGCTTTTCTGTGACTGGTGAGTACTCAACCAAGTCATTCTGAGAATAGTGTATGCGGCGACCGAGTTGCTCTTGCCCGGCGTCAATACGGGATAATACCGCGCCACATAGCAGAACTTTAAAAGTGCTCATCATTGGAAAACGTTCTTCGGGGCGAAAACTCTCAAGGATCTTACCGCTGTTGAGATCCAGTTCGATGTAACCCACTCGTGCACCCAACTGATCTTCAGCATCTTTTACTTTCACCAGCGTTTCTGGGTGAGCAAAAACAGGAAGGCAAAATGCCGCAAAAAAGGGAATAAGGGCGACACGGAAATGTTGAATACTCATATTCTTCCTTTTTCAATATTATTGAAGCATTTATCAGGGTTATTGTCTCATGAGCGGATACATATTTGAATGTATTTAGAAAAATAAACAAATAGGGGTCAGTGTTACAACCAATTAACCAATTCTGAACATTATCGCGAGCCCATTTATACCTGAATATGGCTCATAACACCCCTTGCTCATGACCAAAATCCCTTAACGTGAGTTACGCGCGCGTCGTTCCACTGAGCGTCAGACCCCGTAGAAAAGATCAAAGGATCTTCTTGAGATCCTTTTTTTCTGCGCGTAATCTGCTGCTTGCAAACAAAAAAACCACCGCTACCAGCGGTGGTTTGTTTGCCGGATCAAGAGCTACCAACTCTTTTTCCGAAGGTAACTGGCTTCAGCAGAGCGCAGATACCAAATACTGTTCTTCTAGTGTAGCCGTAGTTAGCCCACCACTTCAAGAACTCTGTAGCACCGCCTACATACCTCGCTCTGCTAATCCTGTTACCAGTGGCTGCTGCCAGTGGCGATAAGTCGTGTCTTACCGGGTTGGACTCAAGACGATAGTTACCGGATAAGGCGCAGCGGTCGGGCTGAACGGGGGGTTCGTGCACACAGCCCAGCTTGGAGCGAACGACCTACACCGAACTGAGATACCTACAGCGTGAGCTATGAGAAAGCGCCACGCTTCCCGAAGGGAGAAAGGCGGACAGGTATCCGGTAAGCGGCAGGGTCGGAACAGGAGAGCGCACGAGGGAGCTTCCAGGGGGAAACGCCTGGTATCTTTATAGTCCTGTCGGGTTTCGCCACCTCTGACTTGAGCGTCGATTTTTGTGATGCTCGTCAGGGGGGCGGAGCCTATGGAAAAACGCCAGCAACGCGGCCTTTTTACGGTTCCTGGCCTTTTGCTGGCCTTTTGCTCACATGTTCTTTCCTGCGTTATCCCCTGATTCTGTGGATAACCGTATTACCGCCTTTGAGTGAGCTGATACCGCTCGCCGCAGCCGAACGACCGAGCGCAGCGAGTCAGTGAGCGAGGAAGCGGAAGGCGAGAGTAGGGAACTGCCAGGCATCAAACTAAGCAGAAGGCCCCTGACGGATGGCCTTTTTGCGTTTCTACAAACTCTTTCTGTGTTGTAAAACGACGGCCAGTCTTAAGCTCGGGCCCCCTGGGCGGTTCTGATAACGAGTAATCGTTAATCCGCAAATAACGTAAAAACCCGCTTCGGCGGGTTTTTTTATGGGGGGAGTTTAGGGAAAGAGCATTTGTCAGAATATTTAAGGGCGCCTGTCACTTTGCTTGATATATGAGAATTATTTAACCTTATAAATGAGAAAAAAGCAACGCACTTTAAATAAGATACGTTGCTTTTTCGATTGATGAACACCTATAATTAAACTATTCATCTATTATTTATGATTTTTTGTATATACAATATTTCTAGTTTGTTAAAGAGAATTAAGAAAATAAATCTCGAAAATAATAAAGGGAAAATCAGTTTTTGATATCAAAATTATACATGTCAACGATAATACAAAATATAATACAAACTATAAGATGTTATCAGTATTTATTATCATTTAGAATAAATTTTGTGTCGCCCTTAATTGTGAGCGGATAACAATTACGAGCTTCATGCACAGTGGCGTTGACTCAATATTGGCCATTAGCCATATTATTCATTGGTTATATAGCATAAATCAATATTGGCTATTGGCCATTGCATACGTTGTATCTATATCATAATATGTACATTTATATTGGCTCATGTCCAATATGACCGCCATGTTGGCATTGATTATTGACTAGTTATTAATAGTAATCAATTACGGGGTCATTAGTTCATAGCCCATATATGGAGTTCCGCGTTACATAACTTACGGTAAATGGCCCGCCTGGCTGACCGCCCAACGACCCCCGCCCATTGACGTCAATAATGACGTATGTTCCCATAGTAACGCCAATAGGGACTTTCCATTGACGTCAATGGGTGGAGTATTTACGGTAAACTGCCCACTTGGCAGTACATCAAGTGTATCATATGCCAAGTACGCCCCCTATTGACGTCAATGACGGTAAATGGCCCGCCTGGCATTATGCCCAGTACATGACCTTATGGGACTTTCCTACTTGGCAGTACATCTACGTATTAGTCATCGCTATTACCATGGTGATGCGGTTTTGGCAGTACATCAATGGGCGTGGATAGCGGTTTGACTCACGGGGATTTCCAAGTCTCCACCCCATTGACGTCAATGGGAGTTTGTTTTGGCACCAAAATCAACGGGACTTTCCAAAATGTCGTAACAACTCCGCCCCATTGACGCAAATGGGCGGTAGGCGTGTACGGTGGGAGGTCTATATAAGCAGAGCTCGTTTAGTGAACCGTCAGATCGCCTGGAGACGCCATCCACGCTGTTTTGACCTCCATAGAAGACACCGGGACCGATCCAGCCTCCGCGGCCGGGAACGGTGCATTGGAACGCGGATTCCCCGTGCCAAGAGTGACGTAAGTACCGCCTATAGAGTCTATAGGCCCACCCCCTTGGCTTCGTTAGAACGCGGCTACAATTAATACATAACCTTATGTATCATACACATACGATTTAGGTGACACTATAGAATAACATCCACTTTGCCTTTCTCTCCACAGGTGTCCACTCCCAGGTCCAACTGCACCTCGGTTCTATCGAAAACGCGCCTGCTAGCGCCACC
